# Ecological Patterns of Gut Microbiota During Elexacaftor/Tezacaftor/Ivacaftor Therapy in Cystic Fibrosis

**DOI:** 10.3390/microorganisms14092053

**Published:** 2026-09-14

**Authors:** Lorenza Putignani, Fabiana Ciciriello, Luigia Turco, Federica Del Chierico, Chiara Marangelo, Matteo Scanu, Francesca Toto, Enza Montemitro, Valentino Bezzerri, Ersilia Fiscarelli, Vincenzina Lucidi, Alessandro Fiocchi, Federico Alghisi, Renato Cutrera

**Affiliations:** 1 Unit of Microbiomics and Unit of Research of Microbiome, Bambino Gesù Children’s Hospital, IRCCS, 00146 Rome, Italy; 2 Department of Life Science, Health and Health Professions, Link Campus University, 00165 Rome, Italy; v.bezzerri@unilink.it; 3 Pneumology and Cystic Fibrosis Unit, Bambino Gesù Children’s Hospital, IRCCS, Piazza Di Sant’Onofrio, 4, 00165 Rome, Italy; fabiana.ciciriello@opbg.net (F.C.); enza.montemitro@opbg.net (E.M.); federico.alghisi@opbg.net (F.A.); 4 Unit of Research of Microbiome, Bambino Gesù Children’s Hospital, IRCCS, 00146 Rome, Italy; luigia.turco@opbg.net (L.T.); federica.delchierico@opbg.net (F.D.C.); chiara.marangelo@opbg.net (C.M.); matteo.scanu@opbg.net (M.S.); francesca.toto@opbg.net (F.T.); 5 Cystic Fibrosis Center, Azienda Ospedaliera Universitaria Integrata, 37126 Verona, Italy; 6 Cystic Fibrosis Microbiology, Bambino Gesù Children’s Hospital, IRCCS, 00146 Rome, Italy; ersilia.fiscarelli@gmail.com; 7 Cystic Fibrosis Unit, Bambino Gesù Children’s Hospital, IRCCS, Piazza Di Sant’ Onofrio, 4, 00165 Rome, Italy; vincenzina.lucidi@gmail.com; 8 Allergy Diseases Research Area, Pediatric Allergology Unit, Bambino Gesù Children’s Hospital, IRCCS, 00146 Rome, Italy; alessandro.fiocchi@allegriallergia.net

**Keywords:** cystic fibrosis (CF), gut microbiota (GM), elexacaftor/tezacaftor/ivacaftor (ETI), microbial ecological perturbations

## Abstract

Cystic fibrosis (CF) is a genetic disorder caused by *CFTR* gene mutations. The elexacaftor/tezacaftor/ivacaftor (ETI) drug combination has markedly improved CF outcomes; however, its impact on gut microbiota (GM) dysbiosis remains poorly investigated. Herein, we examined potential longitudinal effects of ETI treatment on six CF patients’ GM before therapy (T0), and after 6 (T1) and 12 months (T2). Patients’ stools were analyzed using *16S* rRNA metataxonomy, and GM profiling was characterized by alpha diversity, performed using the Shannon–Weiner, Simpson, and Chao1 indices; beta diversity, based on Bray–Curtis distance; and taxonomic patterns through multivariate and univariate analyses. A longitudinal exploratory assessment of the GM composition of CF patients at T0, T1, and T2 was performed. At baseline, CF patients displayed a distinct genus-level microbial signature compared with healthy controls, including *Veillonella_A*, *Haemophilus_D_735815*, *Fusobacterium_C*, *Ruminococcus_B,* and *Blautia_A_141780.* During ETI treatment, alpha diversity showed no significant transient increase at T1 followed by a decrease at T2. Taxonomic profiles showed marked inter-individual heterogeneity. Exploratory longitudinal analyses, assessed by the Friedman test, identified temporal variation in 11 bacterial genera: *CAG-177*, *Clostridium*_*AP*, *Coprococcus*_*A*_121497, *Copromonas*, and *Muricomes*_149725, showed a progressive decrease in relative abundance from T0 to T2; *Haemophilus*_*D*_*734546*, *Eubacterium*_*B*, *Lachnoanaerobaculum*, *Moraxella*_C_651924, and *Neisseria*_*563205* displayed a transient increase at T1, followed by a decrease at T2; and *Lactococcus*_A_346120 showed a transient decrease at T1 followed by an increase at T2. Progressive depletion and transient changes or recovery of bacterial taxa may only represent a rough idea of the ecological changes in the gut microbiota observed during the course of ETI therapy. Longitudinally studies based on large cohorts of CF patients are mandatory to properly interpret these very preliminary results and to search for actual ETI-induced microbiome effects.

## 1. Introduction

Cystic fibrosis (CF) is a life-threatening hereditary disorder that has been estimated to affect 160,000 people worldwide, making it the most lethal genetic condition among Caucasians [1,2]. The disease is caused by mutations in the cystic fibrosis transmembrane conductance regulator (*CFTR*) gene, which encodes the epithelial chloride (Cl^−^) and bicarbonate (HCO_3_^−^) anion channels [3]. Loss of CFTR function decreases cellular Cl^−^ and HCO_3_^−^ secretion, while increasing sodium (Na^+^) absorption [1]. These changes in extracellular ionic concentrations lead to dehydration and alterations of the biophysical properties of the mucus layer, causing it to thicken at the mucosal surfaces of the lungs, digestive tract, and other organs. As a result, people with CF (pwCF) suffer from a variety of symptoms such as airway obstruction, respiratory infections, and malnutrition [4]. Moreover, pwCF suffer from a range of persistent gastrointestinal (GI) complications throughout life, including intestinal dysmotility and hepatopancreatic biliary disease [5].

In the intestinal tract, the mucus layer, primarily composed of mucins (glycoproteins), interacts with the gut microbiota (GM) in multiple ways, coating the epithelial lining and acting as a physical barrier that prevents direct contact between microbes and epithelial cells. Indeed, the outer mucus layer provides a niche for commensals, stimulating mucus secretion by goblet cells and metabolizing mucin glycans as a nutrient source, whereas the inner mucus layer protects host tissue from microbial invasion, promoting commensal tolerance and activating defenses against pathogens. This delicate homeostasis [6] can be perturbed by CF-specific antibiotic therapies, as documented by numerous studies comparing the GM of pwCF with that of healthy subjects [7,8,9,10,11]. The GM of pwCF is characterized by an increase of Enterobacterales, *Streptococcus*, *Veillonella*, and *Staphylococcus* [12]. CF intestinal dysbiosis is also associated with the establishment of chronic inflammation, functional digestive impairment, and increased intestinal permeability [13,14,15,16]. Increasing evidence suggests that intestinal dysbiosis may contribute not only to gastrointestinal manifestations of CF but also to systemic inflammation and disease progression through the gut–lung axis, a bidirectional communication network linking the intestinal and respiratory microbiota [17].

Changes in the composition and metabolic activity of the gut microbiota have been associated with pulmonary outcomes, frequency of exacerbations, nutritional status, and response to therapy [18,19]. Therefore, the intestinal microbiota is recognized as a potential biomarker of disease severity and a promising therapeutic target for microbiome-based interventions aimed at improving clinical outcomes in individuals with CF.

Indeed, clinical trials involving pre- or probiotic treatment have shown amelioration of some CF-related symptoms, suggesting a relationship between GM and CF disease [20,21].

Although CF still significantly reduces life expectancy in the affected population, the introduction of highly effective modulator therapies targeting the CFTR protein has played a large role in improving survival rates of pwCF [22,23]. While many CFTR modulators currently exist, the efficacy of these therapeutic strategies largely depends on the specific type of *CFTR* genetic mutation [24]. The elexacaftor/tezacaftor/ivacaftor (ETI) drug combination has made a significant impact on the survival of pwCF, carrying at least one copy of the F508del *CFTR* mutation, which has been previously excluded from other CFTR modulator therapies [24,25,26,27]. F508del is the most common mutation in CF in the Caucasian population, causing the CFTR protein to misfold and be retained in the endoplasmic reticulum [24]. Elexacaftor and tezacaftor act as correctors, helping the CFTR protein to fold correctly and integrate into the cell membrane. Ivacaftor, on the other hand, acts as a potentiator, helping CFTR to increase the Cl^−^ flow. Together, this triple combination therapy has been shown to increase the function of F508del-CFTR and thus ameliorate symptoms in pwCF [24,25,26,27,28]. ETI therapy was approved by the Food and Drug Administration (FDA) for CF patients aged 12 years and older carrying at least one F508del mutation in 2019 [29]. Subsequently, following a brief period of compassionate use in Europe, ETI was also approved by the European Medicines Agency (EMA) for the treatment of almost 70% of patients with CF [30,31]. Of note, the FDA has recently expanded approval to a panel of 272 *CFTR* variants. In fact, several recent reports have shown that ETI can correct and potentiate additional rare variants, with several clinical benefits specifically for N1303K [32,33,34].

Given the relationship between GM and many CF-related symptoms, both in the gut and in the lungs, there is a growing interest in understanding how CFTR modulation can affect GM composition [35]. A recent study reported restructuring of the lung microbiome following ETI treatment, which correlated with an interesting decrease in CF-related pathogens [36]. In the gut, treatment with tezacaftor and ivacaftor alone had little to no effect on the GM, whereas ETI triple therapy in children reshaped the GM, affecting the intestinal carriage of pathogens associated with CF [37,38]. Here, we describe six CF cases in which, due to advanced disease, ETI treatment was administered under compassionate use before EMA approval. GM longitudinal remodeling and temporal instability was mainly characterized by either progressive depletion of selected bacterial taxa or transient taxonomic shifts.

## 2. Materials and Methods

### 2.1. Patient Enrollment and Sample Collection

Six CF patients, aged 30.7 ± 7.1 (mean ± SD) years and deemed eligible for compassionate use of ETI treatment, were enrolled in a single-center, nonprofit, longitudinal pilot study at the OPBG in Rome, Italy, beginning in April 2020. For microbiota comparisons, 12 healthy control subjects (CTRLs), 6 males and 6 females, were selected from the reference Microbiome Special Collection of OPBG BBMRI biobank, established through an epidemiological survey conducted at the Microbiome Research Unit of Bambino Gesù Children’s Hospital, IRCCS (EUBIOM Project), a node of the Italian Biobanking and Biomolecular Resources Research Infrastructure. The biobank comprises biological samples collected from healthy pediatric subjects and was approved by the Ethics Committee of Bambino Gesù Children’s Hospital (Protocol No. 2839_OPBG_2022; Approval No. 1076, July 26, 2022; CE Version 1.0, 05/09/2022 EUBIOM/2839_OPBG_2022). The CTRLs constituted an independent reference group and were not subjected to the longitudinal follow-up observations. The CTRL subjects were age-matched (33.7 ± 10.6, mean ± SD) to CF patients and had no history of chronic or GI disease. Stool samples and associated metadata were retrieved and processed following the same standardized pipeline for GM metataxonomic analysis of CF patients.

Patients were selected according to a proved CF diagnosis and eligibility for a compassionate use treatment program according to: advanced lung disease (FEV1 < 40%, waiting for lung transplantation, non-invasive ventilation, O_2_ supplementation) and/or a rapid decline in lung function; no alternative therapies available; and ineligibility for clinical trials. Patients experiencing an acute pulmonary exacerbation at the time of enrollment were excluded. Only CF patients were longitudinally sampled for GM analyses. Subject to patient compliance, fecal samples were collected before ETI initiation (T0), 6 months after ETI initiation (T1), and 12 months after ETI initiation (T2). However, some clinical features were also registered at T − 1 (12 months prior to ETI treatment, T0) and T + 1 (24 months after ETI treatment, T0).

After sampling, fecal samples were stored at −80 °C at the OPBG Microbiome Biobank until processing at the Unit of Research of Microbiome.

For GM analyses, samples were classified into four groups: the CTRLs and the CF before ETI treatment (T0); the CF after 6 months of treatment (T1); and after 12 months of treatment (T2). Both longitudinal comparisons among the CF samples at T0, T1, and T2 and case–control comparisons between the CTRL group and the CF patients at T0 were performed.

This study was conducted in accordance with the Declaration of Helsinki, and all procedures were approved by the local Ethics Committee of Bambino Gesù Children’s Hospital (OPBG) (Institutional Review Board, IRB, Protocol No. 911, approved on 22 December 2021). Written informed consent was obtained from all enrolled patients, who were all aged 18 years or older.

### 2.2. Bacterial DNA Extraction and GM Metataxonomy

Bacterial DNA was extracted from 200 mg of 18 stool samples collected at T0, T1, and T2 with a QIAmp Fast DNA Stool mini kit (Qiagen, Hilden, Germany), according to the manufacturer’s instructions. The 3-month time point was not included in the ecological analyses due to incomplete sample availability. The V3–V4 regions of the 16S rRNA gene (~460 bp) were amplified by PCR using the primers 16S_F (5′-TCG TCG GCA GCG TCA GAT GTG TAT AAG AGA CAG CCT ACG GGN GGC WGC AG-3′) and 16S_R (5′-GTC TCG TGG GCT CGG AGA TGT GTA TAA GAG ACA GGA CTA CHV GGG TAT CTA ATC C-3′), following the Illumina MiSeq *16S* rRNA Amplicon Sequencing protocol. PCR reactions were performed using the FastStart High Fidelity Taq Kit (Roche Diagnostics, Mannheim, Germany) with the following cycling conditions: initial denaturation at 95 °C for 3 min; 32 cycles of denaturation at 95 °C for 30 s; annealing at 55 °C for 30 s; and extension at 72 °C for 30 s, followed by a final extension at 72 °C for 5 min.

PCR products were purified using 20 μL of KAPA Pure Beads (Roche Diagnostics, Mannheim, Germany) and subsequently indexed with unique barcode combinations through a second amplification step based on Nextera technology. The resulting libraries were purified, quantified using the Quant-iT^TM^ PicoGreen^®^ dsDNA Assay Kit (Thermo Fisher Scientific, Waltham, MA, USA), and normalized to 4 nM. Libraries were pooled, denatured, and diluted to a final concentration of 7 pM prior to sequencing on an Illumina MiSeq platform (Illumina, San Diego, CA, USA). Paired-end sequencing was performed with a read length of 300 bp.

### 2.3. 16S rRNA Gene Sequencing Data Processing

A total of 36 paired-end FASTQ files were imported into QIIME 2 (version 2024.5) [39]. Sequence quality control was performed using the DADA2 plugin [40], which was employed to denoise reads, remove chimeric sequences, merge paired-end reads, and infer Amplicon Sequence Variants (ASVs). Taxonomic assignment was performed using the Greengenes2 plugin with the Greengenes2 reference database (release October 2022, http://www.greengenes.secondgenome.com (accessed on 12 January 2026)) [41]. The ASV abundance table and taxonomic assignments were imported into R (version 4.3.2) for downstream analyses.

### 2.4. Statistical Analyses

For diversity analyses, the ASV count table was first rarefied to the minimum sequencing depth across samples to account for differences in library size. Alpha diversity was assessed using the Shannon–Wiener, Simpson, and Chao1 indices, and differences between groups were evaluated using the non-parametric Mann–Whitney U test. Beta diversity was calculated based on the Bray–Curtis dissimilarity matrix, and differences in community composition between the patients and CTRLs at T0 were assessed using Permutational Multivariate Analysis of Variance (PERMANOVA), implemented in the adonis2 function of vegan R package, with 999 permutations. Analysis of Similarities (ANOSIM) was also performed as a complementary statistical approach.

For differential abundance analyses, the genus-level relative abundance table was normalized using the Cumulative Sum Scaling (CSS) method [42]. Low-prevalence and low-abundance taxa were filtered by retaining only genera detected in at least 25% of the samples and exhibiting a relative abundance greater than 0.01%. Univariate differential abundance analysis was performed using Linear Discriminant Analysis Effect Size (LEfSe) [43], applying a significance threshold of *p*-value < 0.05 and a logarithmic LDA score cut-off of 3 to identify discriminative bacterial genera. Multivariate analysis was conducted with an exploratory unsupervised approach such as Principal Component Analysis (PCA), which was performed with the prcomp function.

For longitudinal analysis, the non-parametric Friedman test was applied to assess the overall differences across the T0, T1, and T2 time points because of the paired nature of the repeated measurements for each patient, with each of them contributing observations at all three time points in the small size cohort considered. Therefore, alpha diversity indices and genus-level bacterial abundances were statistically considered by this longitudinal approach.

All alpha diversity measures and genus abundance values showing statistically significant differences were subsequently analyzed by Wilcoxon signed-rank test for pairwise comparisons between time points. For beta diversity analysis, PERMANOVA was based on 9999 constrained permutations within subjects, by using the “strata argument” of the adonis2 function, and was applied to the Bray–Curtis dissimilarities with the aim of assessing the differences in community composition across the T0, T1, and T2 time points.

### 2.5. Inference of Microbial Functional Profiles and Differential Pathway Analysis

To predict functional pathways, the Phylogenetic Investigation of Communities by Reconstruction of Unobserved States (PICRUSt2) [44] software was used, exploiting the Kyoto Encyclopedia of Genes and Genomes (KEGG) orthologs database. Differences in the abundance of predicted metabolic pathways between groups were assessed using LEfSe to identify predicted pathways that were differentially represented.

### 2.6. Culture-Based Microbiological Analysis

BAL specimens collected from pwCF were processed according to routine procedures employed in a clinical microbiology laboratory for the detection of respiratory pathogens.

Upon arrival in the laboratory, BAL samples were homogenized by vortexing and quantitatively cultured by inoculating aliquots onto a panel of selective and non-selective media. The samples were plated on Columbia Blood Agar supplemented with 5% sheep blood, Chocolate Agar, MacConkey Agar, Mannitol Salt Agar, Cetrimide Agar, and *Burkholderia cepacia* Selective Agar (BCSA). Plates were incubated at 35–37 °C for 24–48 h under aerobic conditions, with Chocolate Agar incubated in an atmosphere enriched with 5% CO_2_. Cultures on BCSA were maintained for up to 72 h to improve the recovery of slow-growing *Burkholderia* species.

For anaerobic culture, BAL specimens were inoculated onto Schaedler Blood Agar supplemented with 5% sheep blood and selective anaerobic media containing kanamycin and vancomycin when appropriate. Anaerobic cultures were incubated at 37 °C for up to 5–7 days in an anaerobic atmosphere consisting of approximately 85% N_2_, 10% H_2_, and 5% CO_2_.

Following incubation, all morphologically distinct colonies were subcultured when necessary and identified by matrix-assisted laser desorption/ionization time-of-flight mass spectrometry (MALDI-TOF MS; Bruker Daltonics, Bremen, Germany). Antimicrobial susceptibility testing was performed according to routine laboratory procedures and interpreted following current international guidelines.

## 3. Results

### 3.1. Patients’ Clinical Features

Patients included in the study presented heterogeneous genetic backgrounds and clinical characteristics, reflecting the real-world setting of ETI compassionate use, in which therapy is administered to eligible individuals rather than within a predefined, selected homogeneous cohort. Consequently, inter-individual variability may be influenced by multiple host-related factors other than environmental exposures, including genetics, age, and gender.

Patient 1 (P1) was a 27-year-old male patient who was followed at Ospedale Pediatrico Bambino Gesù (OPBG) from birth until age 19, and again from age 29. P1 was diagnosed with CF with pancreatic insufficiency during the early months of life, with a *CFTR* genotype F508del/dele22-24. Despite pancreatic insufficiency, P1 did not present with cystic fibrosis-related diabetes (CFRD) or cystic fibrosis-related liver disease (CFLD), except for the presence of biliary stones. P1 also exhibited a history of recurrent pulmonary exacerbations, mild chronic sinusitis, and pulmonary colonization by *Escherichia coli* and *Proteus mirabilis*. Later, during hospitalization, P1 experienced recurrent pneumothoraxes requiring drainage, one of which was complicated by the development of subcutaneous emphysema, which was resolved after pleural drainage replacement. Chest radiography (CT) revealed diffuse bronchiectasis with mucoid impaction and a large emphysematous bulla occupying almost the entire posterior portion of the left lung. Significant pulmonary function impairment was observed (FEV1 40%). Furthermore, the patient was severely malnourished (BMI 19), necessitating gastrostomy placement in early 2019. Given the complexity of this clinical case, the patient was approved for compassionate use of ETI triple therapy in April 2020 (FEV1 at baseline, 39%).

After treatment commenced, FEV1 increased slightly to 43% and remained stable throughout the study. BMI also improved, allowing for the removal of the gastrostomy tube a few months after initiating ETI therapy. ETI treatment was continued for 12 months, until T2, when the treatment period ended. Lung involvement on CT scans remained substantially unchanged at the one-year follow-up after ETI discontinuation, but the patient no longer required hospitalizations or intravenous antibiotic courses, only sporadic oral antibiotics for mild respiratory exacerbations. Nutritional status gradually improved (BMI measured 12 months after ETI treatment end = 20), reaching 21.5 in 2024.

Patient 2 (P2) was a 33-year-old male with CF, carrying the *CFTR* genotype F508del/Q685PfsX4. Initially, P2 preserved pancreatic function, but since 2018, he has developed clinical and laboratory signs of pancreatic insufficiency, for which he started pancreatic enzyme replacement therapy. Uncomplicated chronic sinusitis, good nutritional status, absence of diabetes, and CFLD with hepatic steatosis but without signs of portal hypertension were reported. Pulmonary involvement included diffuse bronchiectasis and colonization by multidrug-resistant (MDR) *Pseudomonas aeruginosa*. Frequent respiratory exacerbations over the years led to a progressive decline in lung function, with FEV1 < 30%, and the need for non-invasive ventilation (NIV). Consequently, the patient was placed on the lung transplant list in June 2019. In April 2020, the patient started CFTR modulator therapy (ETI) under compassionate use (FEV1 32% at baseline). Clinically, the patient showed a reduction in cough and bronchorrhea, with a discrete improvement in lung function (FEV1: 34% 12 months after ETI initiation). However, during the first year of CFTR modulator therapy, the patient required three hospital admissions for pulmonary exacerbations that necessitated intravenous antibiotics. Lung function remained impaired, albeit improved, and the patient continued to require NIV during sleep. In July 2021, P2 underwent one bilateral lung transplantation with a favorable postoperative course. The patient continues regular follow-up with the transplant team and remains clinically stable. A single fecal calprotectin measurement, performed 1 year after ETI initiation, showed mildly elevated levels (164 µg/g feces).

Patient 3 (P3) was a 26-year-old male CF patient, *CFTR* genotype F508del/N1303, with pancreatic insufficiency, recurrent nasal polyposis, and chronic pulmonary colonization by MDR *P. aeruginosa* and intermittent colonization by *Achromobacter xylosoxidans.* The patient also had significant pulmonary parenchymal damage characterized by bilateral bronchiectasis and right upper lobe consolidation, which persisted after bronchoalveolar lavage. The patient experienced recurrent episodes of hemoptysis and underwent embolization of the right bronchial artery. Over time, the clinical course was characterized by frequent respiratory exacerbations requiring hospitalization and intravenous antibiotic therapy and impairment of pulmonary function, with FEV1 of approximately 46% predicted. The patient was not dependent on supplemental oxygen but presented with suboptimal nutritional status (BMI 17). No evidence of CFLD or CFRD was reported. Due to the severity of the lung disease and the high frequency of hospitalizations, the patient was placed on the lung transplant waiting list in 2016. In April 2020, compassionate use therapy with ETI was initiated (baseline FEV1 45%, BMI 18.5). ETI treatment was continued for 12 months, until T2, when the treatment period ended. Following the start of ETI therapy, the patient showed a marked clinical improvement, including a substantial reduction in respiratory symptoms and pulmonary exacerbations. Between 2020 and 2024, only six courses of intravenous antibiotics were required, compared with approximately one course every three months prior to ETI treatment. Lung imaging also showed improvements on CT scan after one year of treatment (reduced bronchial thickening and mucoid impaction). Improved clinical stability was associated with a significant increase in lung function (FEV1 54% at 24 months after ETI initiation) and stable nutritional status (BMI 18.5). Considering these results, the patient was removed from the lung transplant list in 2021. However, the most recent CT scan (2023) revealed an increase in bronchiectasis. Furthermore, a decline in lung function was noted during recent clinical visits (FEV1 49–54%). For this reason, the patient was recently hospitalized for a new course of intravenous antibiotics. The recent clinical deterioration may be attributed to reduced treatment adherence. No data are available regarding fecal calprotectin measurements before and after ETI therapy.

Patient 4 (P4) was a 32-year-old female with CF and pancreatic sufficiency. Her *CFTR* genotype was F508del/I1234V, and the sweat chloride concentration was 76 mmol/L. The diagnosis of CF was established at 9 years of age due to recurrent respiratory symptoms and growth failure.

Her clinical history was notable for recurrent episodes of pancreatitis. She developed chronic airway colonization with *P. aeruginosa*, *A. xylosoxidans*, and *Scedosporium apiospermum*. A single episode of *Nocardia* infection was documented during puberty. Over time, she experienced progressive and severe lung disease, with a marked decline in pulmonary function, reaching FEV1 of 40% predicted. One cycle/month of intravenous antibiotic therapy was prescribed and required hospitalization. Moreover, P4 had frequent episodes of hemoptysis, a lung clearance index (LCI) of 14.4, a poor nutritional status (BMI 20), and impaired glucose tolerance without insulin therapy. She also presented episodes of Distal Intestinal Obstruction Syndrome (DIOS). She underwent functional endoscopic sinus surgery early for severe sinusitis. Given the presence of all risk factors, the patient was placed on the lung transplant list. In April 2020, she started compassionate use therapy with ETI (baseline FEV1 42%). ETI treatment was continued for 12 months, until T2, when the treatment period ended.

In this patient, we observed improvements in the sweat test (Cl 65 mmol/L) and pulmonary function tests, with rapid increases in FEV1 (60%) and LCI (9.8). The CT scan showed a reduction in bronchial thickening and mucoid impaction. She was not readmitted to the hospital. She was removed from the lung transplant list. The Cystic Fibrosis Questionnaire-Revised showed improvements in the digestive and respiratory domains.

Patient 5 (P5) was a 23-year-old female CF patient with pancreatic insufficiency and CFTR-related liver disease, *CFTR* genotype F508del/621 + 1G > T, sweat test Cl 60 mmol/L, and pulmonary colonization by *Burkholderia cepacia*, *P. aeruginosa*, and *Staphylococcus aureus*. Appendectomy at 9 years complicated by an intestinal abscess required resection of 10 cm of intestine and then ileo-colic anastomosis. Then, histological examination diagnosed Crohn’s disease. After that, the patient experienced recurrent episodes of DIOS and underwent severe weight loss (BMI measured 12 months before starting treatment = 19). The patient received numerous cycles of intravenous antibiotic therapy for which an intrathoracic CVC was placed. She had two episodes of massive hemoptysis that required angiography with subsequent bronchial artery embolization, without complications. P5 underwent numerous breathing tests, which showed a rapid decline in lung function during the year preceding ETI initiation, with reduction in FEV1 predicted values from 48% to 38% at T − 1. Considering clinical worsening, the patient started ETI under compassionate use in April 2020 (FEV1 46% at baseline). After only 3 days of treatment, FEV1 further increased to 58% and, subsequently, the patient showed rapid clinical improvement, with reduced respiratory symptoms and no fever, as well as weight gain (BMI measured 12 months after ETI treatment end = 22). Cough resolved after treatment and pulmonary function improved, with FEV1 reaching 77% 24 months after therapy initiation (T + 1), while the LCI decreased from 16 to 7.5. The patient also showed an improvement in exercise capacity, with the 6-min Walk Test (6MWT) distance increasing from 450 m to 600 m. CT scan showed multiple bronchiectases and large areas of air-trapping and mucoid impaction of the bronchi.

Chest CT scan showed reduced bronchial wall thickening and mucoid impaction. The patient did not require hospitalization during the observation period. The sweat chloride test measured 55 mmol/L. In addition, fecal calprotectin levels normalized after the initiation of ETI therapy, whereas they had been elevated prior to treatment.

Patient 6 (P6) was a 43-year-old male CF patient with pancreatic insufficiency. The patient carried the *CFTR* genotype F508del/W1282X and had a positive sweat test with chloride levels of 84 mmol/L. The clinical course was characterized by severe respiratory insufficiency requiring oxygen therapy, with an FEV1 of 34% predicted. The patient also presented with CFLD and CFRD requiring insulin therapy. Acoustic radiation force impulse elastography (ARFI) revealed moderate hepatic steatosis. The patient had a history of pulmonary colonization with methicillin-resistant *S. aureus* (MRSA), *Stenotrophomonas maltophilia*, *Achromobacter denitrificans*, *Sphingomonas spiritivorum*, and *P. aeruginosa*. Episodes of DIOS occur frequently after puberty. Bone mineral density assessment showed severe osteopenia, with a markedly reduced T-score. The patient also reported recurrent joint pain. At the time of reporting, the patient had been followed for ten years at the Padua Center for potential lung transplantation.

The CT scan showed increased thickening of the bronchial walls; numerous bilateral, ubiquitous, cylindrical bronchiectasis and bronchiectasis; and parenchymal areas of paraseptal emphysema. The scans at maximum expiration show extensive air trapping in the lower lobes. Mucosal thickening of both maxillary sinuses, of chronic appearance, particularly evident on the left, of some ethmoidal cells, and of the frontal sinuses.

P6 started CFTR modulator therapy (ETI) under compassionate use in April 2020 (FEV1 35% at baseline). ETI treatment was continued for 12 months, until T2, when the treatment period ended. Pulmonary function tests were stable, with minimal improvement; quality of life improved, and there were no cough, fever, or fatigue. The patient did not meet the inclusion criteria for lung transplantation. The CT scan also showed improvement in mucoid impaction. The 6MWT also improved. Fecal calprotectin levels were normal after starting ETI, and the sweat test Cl resulted in 62 mmol/L. He still needed cycles of oral antibiotic therapy, but he stopped IV therapy.

Baseline and longitudinal anamnestic and clinical characteristics of the patients are summarized in Table 1 with the intent to describe exploratory clinical and lifestyle confounders.

### 3.2. Ecology, Metataxonomy, and Predicted Functional Pathways of CF Patients’ Gut Microbiota During ETI Treatment

The *16S* rRNA gene sequencing enabled profiling of the fecal microbiota of CF patients treated with ETI. After sequence denoising, trimming, and chimera removal, an average of 58,039 reads per sample was obtained (minimum frequency: 2956 reads). Overall, 4124 Amplicon Sequence Variants (ASVs) were identified.

To characterize the GM profiling of CF patients, we first compared healthy subjects, considered as controls (CTRLs), with patients at baseline, prior to ETI initiation (T0). Alpha diversity, exploited using the Shannon–Weiner, Simpson, and Chao1 indices did not reveal statistically significant differences between the CTRL and CF groups at T0 (Supplementary Figure S1A–C). Similarly, beta diversity, based on Bray–Curtis distance, did not reveal a significant separation between groups in the PCoA plot (Supplementary Figure S1D), as supported by PERMANOVA (R^2^ = 0.0542, *p*-value > 0.05) and ANOSIM (R = −0.0907, *p*-value > 0.05) analyses.

**Figure 1:**
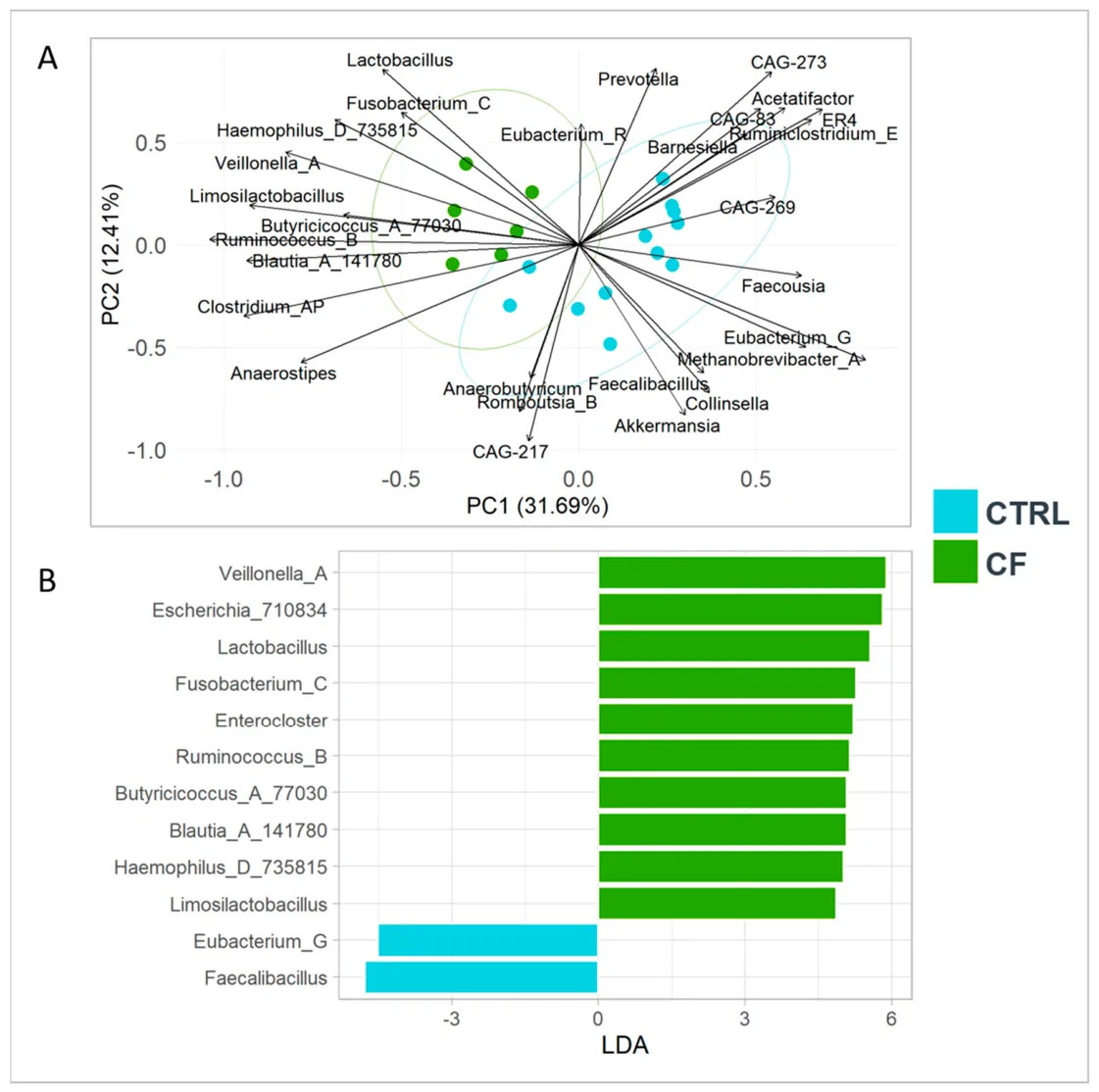
Distinct genus–level microbial signatures characterize CF patients at baseline (T0) compared with healthy subjects considered as the controls (CTRLs). Principal Component Analysis (PCA) showed the separation between groups and identified the taxa contributing most to PC1 and PC2 (31.69% and 12.41% explained variance, respectively) (**A**). Univariate LEfSe analysis identified significantly different taxa between groups (Linear Discriminant Analysis (LDA) > 3; adjusted *p*-value < 0.05) (**B**). Green and blue bars refer to the CF patient and CTRL subject groups, respectively.

To further investigate differences in GM composition at the genus level (L6) between CF patients prior to ETI treatment (T0) and the CTRLs, we applied multivariate and univariate approaches (Figure 1). The multivariate exploratory analysis based on PCA revealed distinct microbial profiles between the two groups. Specifically, *Anaerostipes*, *Clostridium_AP*, *Blautia_A_141780*, *Ruminococcus_B*, *Butyricicoccus_A_77030*, *Limosilactobacillus*, *Veillonella_A*, *Haemophilus_D_735815*, *Fusobacterium_C*, and *Lactobacillus* were the most discriminatory taxa in the GM of the CF patients, while *Anaerobutyricum*, *Romboutsia_B*, *Faecalibacillus*, *Collinsella*, *Akkermansia*, *Methanobrevibacter_A*, *Eubacterium_G*, *Faecousia*, *CAG-269*, *Ruminiclostridium_E*, *ER4*, *CAG-83*, *Barnesiella*, *Acetatifactor*, *CAG-273*, and *Prevotella* showed the highest loadings in the GM of the CTRLs (Figure 1A). The univariate analysis further supported this distinction, identifying *Veillonella_A*, *Haemophilus_D_735815*, *Anaerostipes*, *Fusobacterium_C*, *Ruminococcus_B*, *Butyricicoccus_A_77030*, *Lactobacillus*, and *Blautia_A_141780* among the most abundant taxa in the GM of the CF patients, and *Faecalibacillus* and *Eubacterium_G* as the least represented taxa compared with the CTRLs (Figure 1B).

Still at T0, before ETI treatment, predicted functional profiling based on 16S rRNA gene sequencing was inferred using the Phylogenetic Investigation of Communities by Reconstruction of Unobserved States (PICRUSt2), version 2.4.1. The analysis suggested differences in the predicted gut microbial metabolic pathways between the CF and CTRL groups, with, as a noteworthy observation, higher predicted contributions of the “butanoate metabolism” and “ascorbate and aldarate metabolism” pathways in the GM of CF patients compared with the CTRLs (Supplementary Figure S2). These findings reflect predicted microbial functions based on taxonomic composition and do not represent direct measurements of microbial metabolic activity.

To provide a longitudinal exploratory ecological profiling of CF patients at T0, T1, and T2, GM profiles of the CF patients treated with ETI were produced. Alpha diversity was evaluated using the Shannon–Wiener, Chao1, and Simpson indices (Table 2), while beta diversity, assessed using PERMANOVA with constrained permutations on the Bray–Curtis dissimilarity matrix, did not reveal significant separation of GM composition across time points. Inter-individual dispersion was observed, particularly at T2 (Supplementary Figure S3). Patient 2 and Patient 5 displayed the most evident separation between T0 and T2. Patient 2 showed a progressive shift of the microbial community arrangement at T1 and T2 compared with the baseline composition, whereas patient 5 showed a relatively homogeneous GM profile between T0 and T1, followed by a moderate shift at T2 (Supplementary Figure S3).

**Figure 2:**
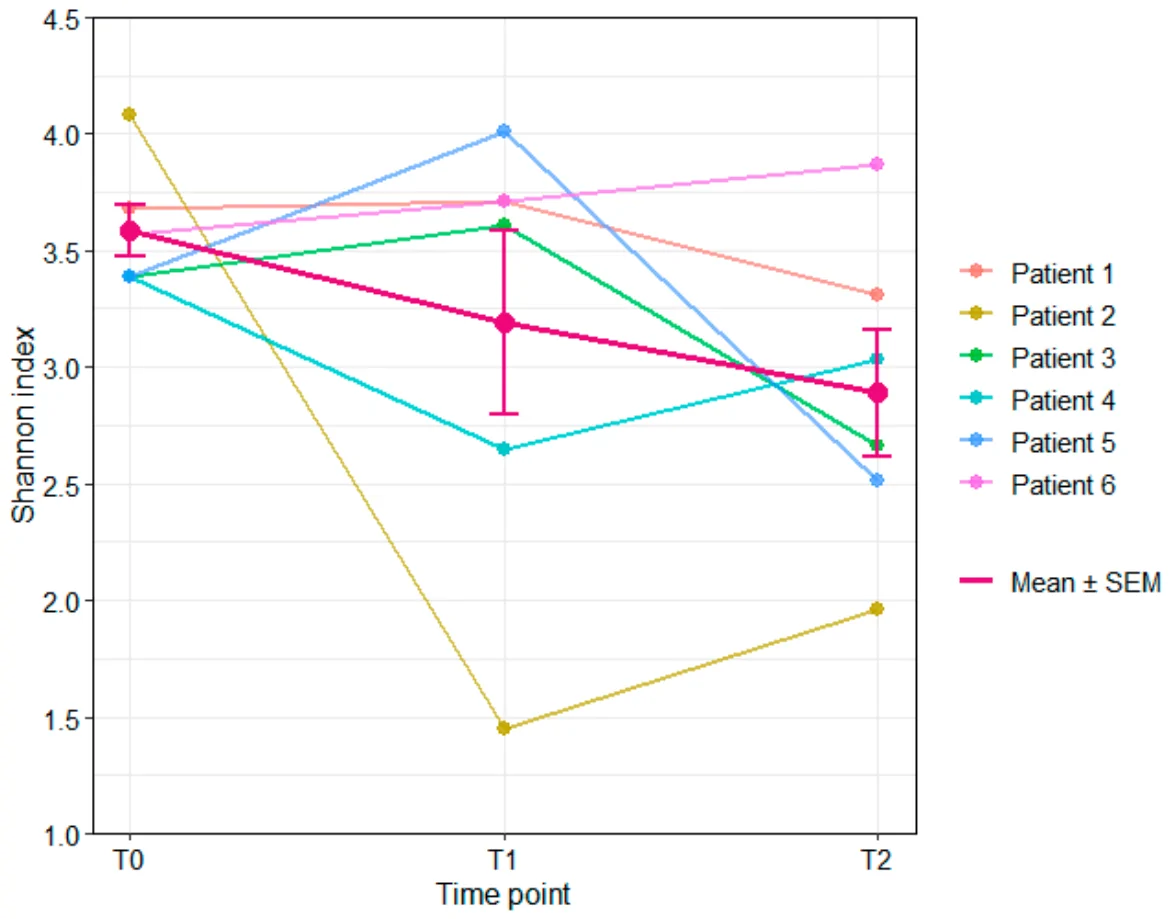
Spaghetti plot of the Shannon–Weiner indices from the longitudinal analysis describing the α diversity of each patient at the T0, T1, and T2 time points. T0 (baseline), T1 (6 months post-ETI), and T2 (12 months post-ETI). Also, mean values and Standard Error of the Mean (SEM) are reported.

To further improve the longitudinal ecological profiling of CF patients at T0, T1, and T2, GM profiles of the CF patients were analyzed by the Friedman test (Supplementary Table S1). Hence, the Shannon–Weiner and Chao1 indexes and Observed ASV abundancies were investigated to determine diversity and taxonomic changes across time. Alpha diversity, focusing on the Shannon–Weiner index, showed no significant decrease at T2 compared to T0 and T1 (median Shannon–Weiner index: 3.58 at T0, 3.19 at T1, and 2.89 at T2; Friedman test, χ^2^ = 1.00, *p*-value = 0.607), as well as Chao1 values (235.69, at T0, 235.73 at T1, and 162.57 at T2; Friedman test, χ^2^ = 2.33, *p*-value = 0.31) (Figure 2).

As expected, pairwise Wilcoxon signed-rank testing confirmed the absence of significant statistics, underlying a trend toward lower Shannon–Weiner diversity at T2 compared with T0 (*p*-value = 0.0625), and lower Chao1 diversity at T2 compared with T0 (*p*-value *p* = 0.24) (Supplementary Table S1). The effect between T0 and T2 could be regarded as considerable, although the presence of only six patients impeded the conventional significance threshold from being reached, hence conferring only an exploratory level of the investigation herein.

**Figure 3:**
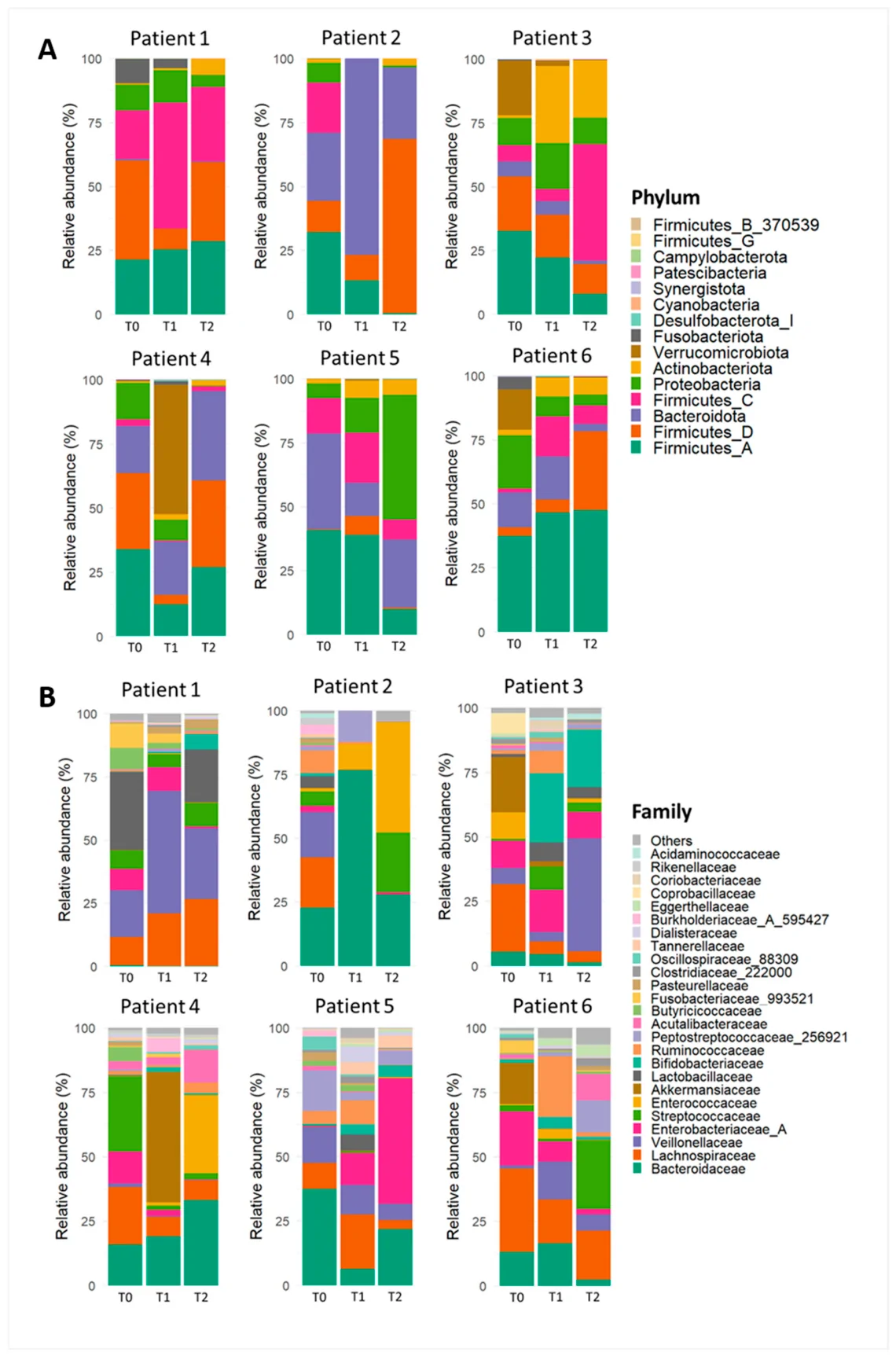
CF patients’ overall GM composition at the phylum (**A**) and family (**B**) levels (L2 and L5). Stacked bar plots represent the phylum– and family–level composition of the GM for each patient at T0, T1, and T2, based on ASV relative abundances. Each bar represents an individual sample, and the colors indicate the bacterial phylum and family, respectively.

**Figure 4:**
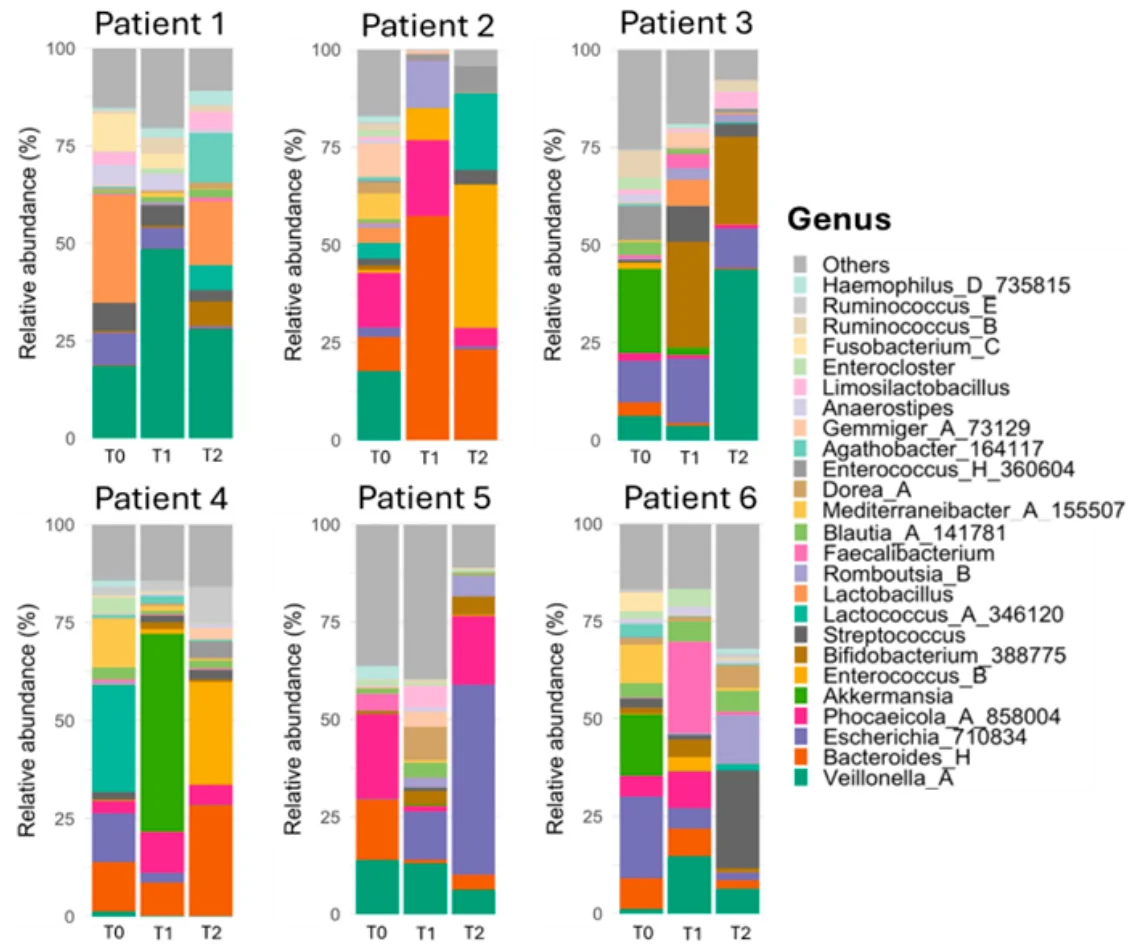
CF patients’ overall GM composition at the genus level (L6). Stacked bar plots represent the genus–level composition of the GM for each patient at T0, T1, and T2, based on ASV relative abundances. Each bar represents an individual sample, and the colors indicate the bacterial genera.

Relative abundances of ASVs for the CF patients at T0, T1, and T2 at the phylum, family (Figure 3), and genus (Figure 4) levels were provided. The relative abundance profiles suggested a certain degree of inter-individual heterogeneity across the three taxonomic levels. At the phylum level, Bacteroidota, Firmicutes, and Proteobacteria seemed to be the most represented phyla, while Bacteroidaceae, Lachnospiraceae, Veillonellaceae, Enterobacteriaceae_A, Streptococcaceae, and Enterococcaceae were the families that most contributed to the individual profiles. At the genus level, *Veillonella_A*, *Bacteroides_H*, *Escherichia_710834*, *Akkermansia*, and *Enterococcus_B* appeared to be the most abundant genera, although their relative distribution varied across patients. Moreover, for each patient, the relative abundance profiles appeared to considerably vary across the three time points, showing different patterns of taxonomic distribution along the T0–T2 time course.

**Figure 5:**
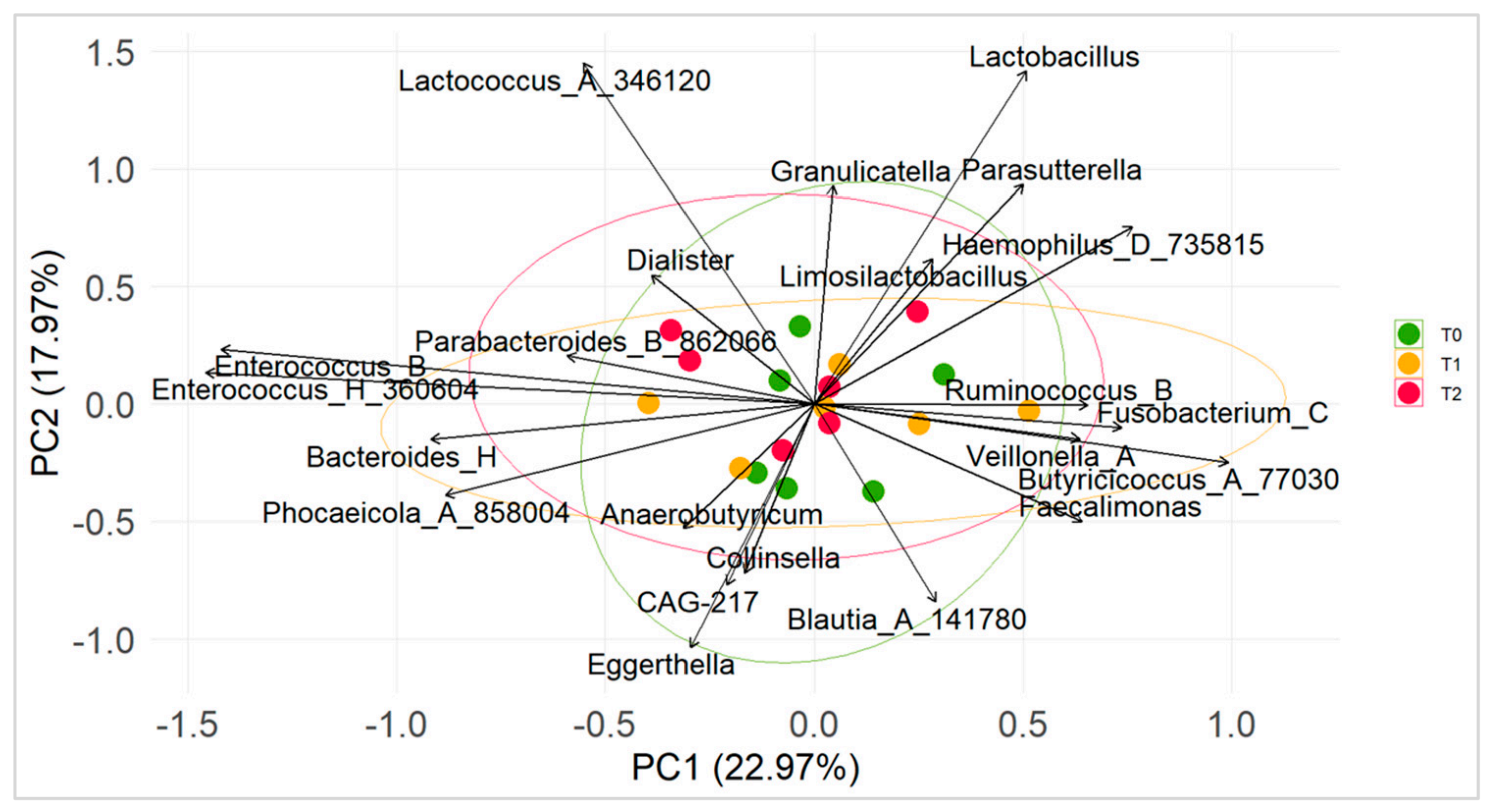
PCA biplot of the longitudinal comparison of the GM profiles among T0, T1, and T2 in CF patients treated with ETI.

To further explore the contribution of taxa to the overall variability in GM composition, an exploratory PCA was subsequently performed on bacterial distribution at the genus level through the time course of T0–T2. The principal loadings contributing to the first two principal components were: *Enterococcus_H_360604*, *Enterococcus_B*, *Butyricicoccus_A_77030*, *Bacteroides_H*, *Phocaeicola_A_858004*, *Haemophilus_D_735815*, *Fusobacterium_C*, *Ruminococcus_B*, *Faecalimonas*, *Veillonella_A*, *Parabacteroides_B_862066*, *Lactococcus_A_346120*, *Lactobacillus*, and *Parasutterella* (Figure 5).

**Figure 6:**
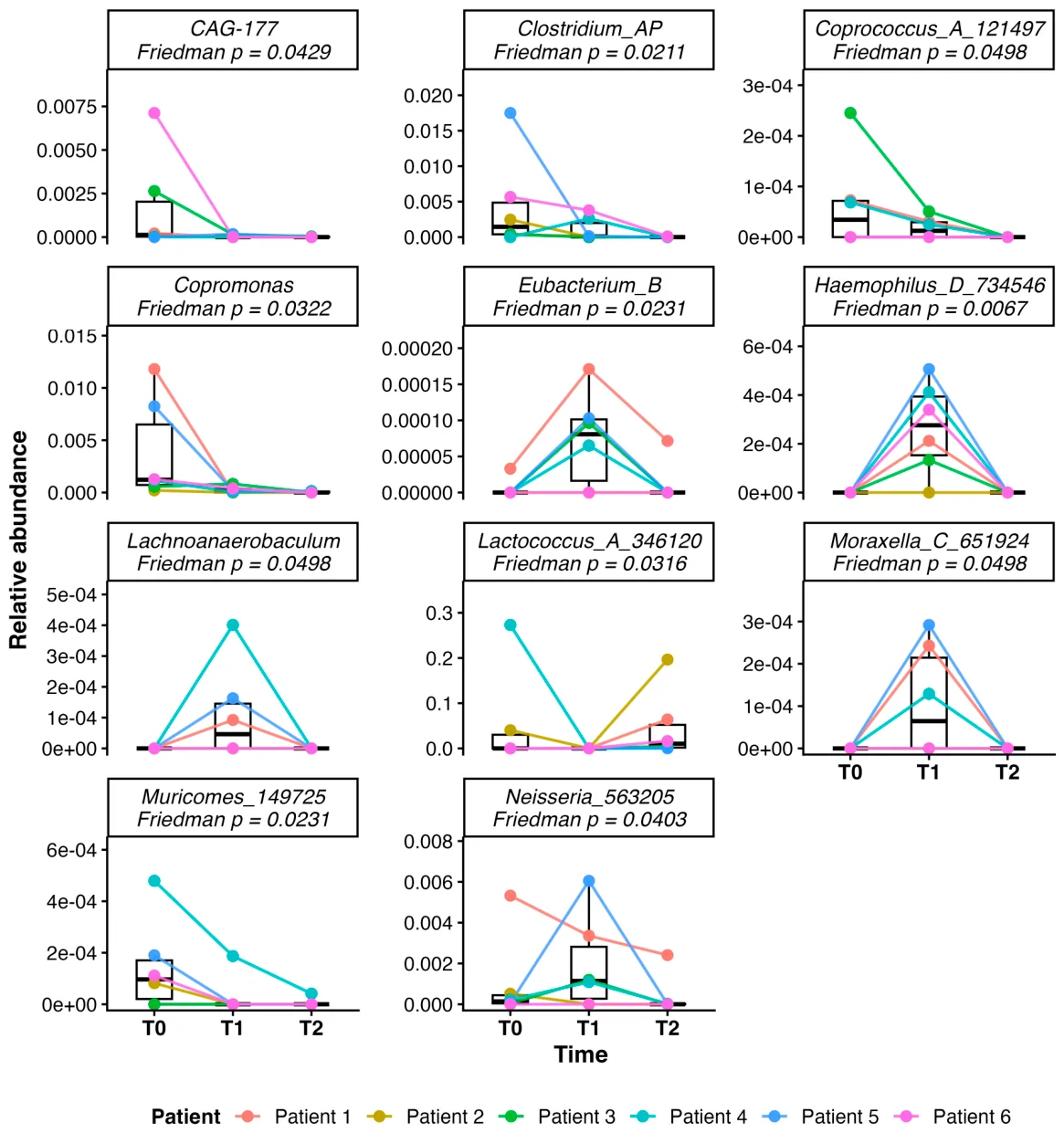
Relative abundances of statistically significant bacterial genera based on the Friedman test, reported for each FC patient analyzed along the time course of T0–T2. Box plots indicate median and quartile values for each time point.

Longitudinal analysis of GM profiling, performed by the Friedman test, identified significant differences in the relative abundance of 11 bacterial genera across T0, T1, and T2: *Haemophilus*_*D*_734546 (*p*-value = 0.0067), *Clostridium*_*AP* (*p*-value = 0.0211), *Eubacterium_B* (*p*-value = 0.0231), *Muricomes*_149725 (*p*-value = 0.0231), *Lactococcus_A*_346120 (*p*-value = 0.0316), *Copromonas* (*p*-value = 0.0322), *Neisseria*_563205 (*p*-value = 0.0403), *CAG*-177 (*p*-value = 0.0429), *Coprococcus*_*A*_121497 (*p*-value = 0.0498), *Lachnoanaerobaculum* (*p*-value = 0.0498), and *Moraxella*_*C*_651924 (*p*-value = 0.0498) (Figure 6, Supplementary Table S4).

Post hoc pairwise comparisons, using the Wilcoxon signed-rank test, identified few taxa, including *CAG*-177, *Clostridium*_*AP*, *Coprococcus*_*A*_121497, *Copromonas*, and *Muricomes*_149725, showing an overall decrease in relative abundance from T0 to T2, providing the first group of progressively depleted taxa. *Haemophilus*_*D*_734546, *Eubacterium*_*B*, *Lachnoanaerobaculum*, *Moraxella*_*C*_651924, and *Neisseria*_563205 displayed, instead, a transient increase at T1, followed by a decrease at T2. Besides these two groups, *Lactococcus*_A_346120 exhibited a drop-out at T1 and a further statistically significant increase a T2 (Figure 6, Supplementary Table S5).

## 4. Discussion

Intestinal dysbiosis is a common feature in pwCF. CFTR dysfunction appears to drive GM imbalance. Therefore, CFTR modulators should be investigated for their effects on the GM. In our study, we focused on the impact of the ETI triple combination on GM diversity and composition in six pwCF with advanced lung disease, assessed before treatment and after 6 and 12 months of therapy, with the primary aim of determining whether ETI treatment longitudinally promoted transient ecological and taxonomic shifts.

First, we investigated whether baseline (T0) GM diversity of pwCF differed from that in healthy subjects. We found no statistically significant differences in diversity and dissimilarity, although we identified distinct taxonomic patterns, with increased representation of genera such as *Veillonella*_A, *Haemophilus*_D_735815, *Ruminococcus*_B, and *Fusobacterium*_C, consistent with the previous literature [10,18]. In particular, the increased relative abundance of the genus *Veillonella* has been extensively reported among CF patients and is considered part of the core respiratory microbiota in CF [45]. At the intestinal level, *Veillonella* is associated with an inflammatory state and is a key bacterial biomarker, providing critical insights into the pathophysiology of CF [8,45,46,47,48,49]. CF patients exhibit profound intestinal dysbiosis driven by CFTR dysfunction and extensive antibiotic exposure, creating a permissive environment for pathogens such as *Clostridioides difficile* [50].

The GM diversity of CF patients at 6 months (T1) and 12 months (T2) post-treatment was characterized by an ecological transient increase at T1, followed by a decrease at T2, as reported for both the Shannon–Weiner and Chao1 values. However, in terms of taxa profiling after 12 months of ETI treatment, a significant difference across time was observed for GM composition. CAG-177, *Clostridium*_*AP*, *Coprococcus_A_121497*, *Copromonas,* and *Muricomes_149725* were characterized by an overall decrease in relative abundance from T0 to T2, until a progressive depletion through the ETI treatment timeline occurred. Notably, several of these taxa belong to phylogenetically related anaerobic lineages within the class Clostridia: *Coprococcus*, *Copromonas*, and *Muricomes* are members of the order of Lachnospiraceae, which is associated with SCFA-producing gut communities, whereas *CAG-177* belongs to the order of Oscillospirales. This taxonomic convergence could suggest transient ecological and taxonomic redistribution of a small part of GM composition, as observed by Marsh et al., with respect to gradual changes in core microbiota diversity in patients with CF after ETI treatment [51].

*Haemophilus_D_734546*, *Eubacterium_B*, *Lachnoanaerobaculum*, *Moraxella_C_651924*, and *Neisseria_563205* were characterized by an interesting transient increase at T1, followed by a decrease at T2, suggesting two GM core communities with potentially different responses to ETI. Apparently, *Lactococcus*_*A_346120* was characterized by a significant increase at the end of ETI treatment at T2 compared to T1.

The genus *Lactococcus* is well known as a probiotic [52,53] and has also been shown to correlate negatively with TGF-β1, a cytokine associated with fibrosis [54]. Oral administration of *L. lactis* has been shown to enhance lung immune responses, providing protection against murine parainfluenza virus infection [55]. However, in this context, the transient enrichment of *Lactococcus*_*A_346120* associated with ETI exposure after 12 months may only reflect microbiota shifts, and possible causal relationships remain to be established next, as our study is still at an exploratory level.

Nevertheless, these results should be interpreted with consideration of important inherent limitations. Indeed, GM composition of pwCF may be influenced by multiple interacting factors, including disease severity, recurrent hospitalizations, environmental exposures, cumulative antibiotic exposure, lifestyle, dietary habits, age, gender, and *CFTR* alterations, which act as GM confounding factors. However, they cannot be fully accounted for in this study due to the small sample size. Consequently, the longitudinally statistical results should be corroborated by a large cohort, suggesting the need of additional research to confirm these very preliminary results.

## 5. Conclusions

In conclusion, our longitudinal findings show that ETI therapy could be associated with a dynamic remodeling of the gut microbial ecosystem, characterized by temporal fluctuations in ecological and taxonomic changes in the GM composition of pwCF who exhibit severe clinical presentations. In particular, the progressive depletion of several bacterial taxa and transient changes or recovery at later time points support the concept that CFTR modulation may be accompanied by a gradual redistribution of microbial communities, suggesting the GM as an interesting ecological trait during ETI therapy.

## Figures and Tables

**Table 1:** Anamnestic and clinical features of patients treated with ETI. Feature values are reported at three times related to ETI treatment: T0 (baseline), T1 (6 months post-ETI), and T2 (12 months post-ETI). The notation T − 1 refers to values collected during 12 months before the start of ETI treatment (T0), whereas T + 1 indicates clinical values collected 24 months after starting ETI treatment (T0). Patient age refers to the age at the first sampling for profiling of GM. Pathogens refers to microorganisms recovered from bronchoalveolar lavage (BAL) samples and identified by targeted culturomics.

	Patient 1	Patient 2	Patient 3	Patient 4	Patient 5	Patient 6
**Gender**	Male	Male	Male	Female	Female	Male
**Age (years)**	27	33	26	32	23	43
**BMI T − 1**	19	22	17	20	19	25
**BMI T0**	19	22	18.5	20.5	18	25
**BMI T + 1**	20	23	18.5	20.5	22	25
**Genotype**	F508del/ CFTRdele22-24	F508del/ Q685PfsX4	F508del/ N1303K	F508del/ I1234V	F508del/ 621 + 1G > T	F508del/ W1282X
**Pathogens T − 1**	*E. coli* *P. mirabilis*	*P. aeruginosa* MDR	*P. aeruginosa* MDR *A. xylosoxidans*	*P. aeruginosa* *A. xylosoxidans* *S. aureus*	*B. cepacia* *P. aeruginosa* *S. aureus*	*S. aureus* MRSA *S. maltophilia* *A. denitrificans* *S. spiritivorum* *P. aeruginosa*
**Pathogens T0**	*E. coli* *P. mirabilis*	*P. aeruginosa* MDR	*P. aeruginosa* MDR *A. xylosoxidans*	NA	*B. cepacia* *S. aureus*	*S. aureus* MRSA *S. maltophilia* *P. aeruginosa*
**Pathogens T + 1**	*E. coli* *P. mirabilis*	Transplant	NA	*P. aeruginosa* *A. xylosoxidans* *S. aureus*	*B. cepacia* *S. aureus*	*P. aeruginosa* *S. aureus* MRSA
**Repeated course of intravenous antibiotics**	Yes	Yes	Yes	Yes	Yes (prior ETI)	Yes
**Repeated course of oral antibiotics**	Yes *	Yes *	Yes *	Yes *	Yes *	Yes *
**PERT**	Yes	Yes	Yes	Yes	Yes	Yes
**Major comorbidities**	NA	CFLD	NA	Recurrent pancreatitis DIOS	Crohn’s disease DIOS	CFLD CFRD
**FEV1 (L/%) T − 1**	1.80 (40)	1.26 (27)	1.65 (46)	1.23 (40)	1.57 (38)	1.26 (34)
**FEV1 (L/%) T0**	1.77 (39)	1.50 (32)	1.60 (45)	1.29 (42)	1.78 (46)	1.31 (35)
**FEV1 (L/%) T1**	1.91 (43)	1.54 (33)	1.61 (45)	1.51 (49)	1.84 (61)	1.15 (32)
**FEV1 (L/%) T2**	1.83 (41)	1.58 (34)	1.62 (43)	1.86 (60)	1.87 (62)	1.20 (33)
**FEV1 (L/%) T + 1**	1.98 (44)	1.59 (34)	1.90 (54)	NA	2.06 (77)	1.24 (34)
**Respiratory failure**	Severe	severe	Severe	Moderate	Moderate	Severe
**Lung transplantation**	*NA*	Transplantation (after ETI)	Transplant candidate (prior to ETI)	Transplant candidate (prior to ETI)	*NA*	Transplant candidate (prior to ETI)

ETI, elexacaftor/tezacaftor/ivacaftor; FEV1, forced Expiratory Volume in 1 s; MDR, multidrug-resistant; MRSA, methicillin-resistant *Staphylococcus aureus*; CFLD, cystic fibrosis-related liver disease; NA, not applicable; DIOS, Distal Intestinal Obstruction Syndrome; CFRD, cystic fibrosis-related diabetes; and PERT, pancreatic enzyme replacement therapy. * Ciproxin OS in the case of mild upper respiratory tract symptoms.

**Table 2:** Values of alpha diversity to describe the ecology of CF patients at the T0, T1, and T2 time points. Alpha diversity is based on the Chao1, Shannon–Weiner, and Simpson indices. T0 (baseline), T1 (6 months post-ETI), and T2 (12 months post-ETI).

Patient ID	Time Point	Chao1	Shannon–Weiner	Simpson
Patient 1	T0	262.19	3.68	0.96
Patient 1	T1	272.91	3.71	0.96
Patient 1	T2	118.75	3.31	0.94
Patient 2	T0	230.81	4.08	0.97
Patient 2	T1	10	1.45	0.69
Patient 2	T2	120.67	1.96	0.79
Patient 3	T0	218.12	3.39	0.93
Patient 3	T1	378.49	3.61	0.93
Patient 3	T2	152.25	2.66	0.84
Patient 4	T0	416.72	3.39	0.90
Patient 4	T1	307.76	2.65	0.74
Patient 4	T2	197.5	3.03	0.89
Patient 5	T0	101.33	3.39	0.94
Patient 5	T1	292.91	4.01	0.96
Patient 5	T2	89.27	2.51	0.85
Patient 6	T0	184.95	3.57	0.93
Patient 6	T1	152.33	3.71	0.96
Patient 6	T2	297	3.87	0.94

## Data Availability

The original contributions presented in this study are included in the article/Supplementary Materials. Further inquiries can be directed to the corresponding authors.

## Supplementary Materials

The following supporting information can be downloaded at: https://www.mdpi.com/article/10.3390/microorganisms14092053/s1, Supplementary Table S1. Values of Friedman and Wilcoxon signed-rank tests performed on GM alpha diversity indices of CF patients at the T0, T1, and T2 time points to assess longitudinal and related pairwise comparisons; Supplementary Table S2. Relative abundance of bacterial families across all analyzed samples. ASVs were taxonomically assigned and collapsed at the family level. The table reports the relative abundance values for each family in each individual sample belonging to the CTRL, T0, T1, and T2 groups, providing an overview of the taxonomic distribution and inter-individual variability of the GM across the study groups and time points; Supplementary Table S3. Relative abundance of bacterial genera across all analyzed samples. ASVs were taxonomically assigned and collapsed at the genus level. The table reports the relative abundance values for each genus in each individual sample belonging to the CTRL, T0, T1, and T2 groups, allowing visualization of genus-level microbial composition and variability among samples and experimental groups; Supplementary Table S4. Longitudinal values of the Friedman test performed on the relative distribution of GM bacterial taxa at level L6 (genus) of CF patients at the T0, T1, and T2 time points; Supplementary Table S5. The statistically significant values of the Wilcoxon signed-rank tests performed on GM ASV relative distributions at the genus level (L6) of CF patients by the Friedman test for pairwise comparison at T0–T1, T1–T2, and T0–T2. Supplementary Figure S1. Ecological comparison between CF patients at baseline (T0) and the healthy controls (CTRLs). No significant differences in alpha diversity were observed by exploiting the Shannon–Weiner, Simpson, and Chao1 indices (A–C). Similarly, beta diversity, based on Bray–Curtis distance, did not reveal a significant separation between groups in the PCoA plot (D) (PERMANOVA: R^2^ = 0.0542, *p*-value > 0.05; ANOSIM: R = −0.09, *p*-value > 0.05); Supplementary Figure S2. Functional prediction identified metabolic pathways specific to CF patients before treatment (T0), compared with healthy controls (CTRL). Functional prediction was performed to identify distinct pathway signatures assigned to pwCF at T0 (green) and CTRL group (blue) using PICRUSt2 and KEGG pathway annotation. Statistics was carried out by applying LEfSe analysis (LDA > 3); Supplementary Figure S3. Longitudinal comparison of gut microbiota profiles among the T0, T1, and T2 time points in CF patients treated with ETI. Beta diversity was assessed using the Bray–Curtis dissimilarity matrix and visualized by PCoA.

## Abbreviations

The following abbreviations are used in this manuscript:

ASVs	Amplicon sequence variants
BAL	Bronchoalveolar lavage
CF	Cystic fibrosis
CFLD	Cystic fibrosis-related liver disease
CFRD	Cystic fibrosis-related diabetes
CFTR	Cystic fibrosis transmembrane conductance regulator
CT	Chest radiography
CTRLs	Controls
DIOS	Distal intestinal obstruction syndrome
EMA	European Medicines Agency
ETI	Elexacaftor/tezacaftor/ivacaftor
FDA	Food and Drug Administration
GI	Gastrointestinal
GM	Gut microbiota
KEGG	Kyoto Encyclopedia of Genes and Genomes
LCI	Lung clearance index
LDA	Linear discriminant analysis
LEfSe	Linear discriminant analysis effect size
MDR	Multi-drug resistant
MRSA	Methicillin-resistant *Staphylococcus aureus*
NIV	Non-invasive ventilation
OPBG	Ospedale Pediatrico Bambino Gesù
PCA	Principal component analysis
PCoA	Principal coordinate analysis
PERT	Pancreatic enzyme replacement therapy
PICRUSt2	Phylogenetic Investigation of Communities by Reconstruction of Unobserved States
pwCF	People with cystic fibrosis
RMSE	Root mean square error
SD	Standard deviation
SEM	Standard error of mean
