# Supplementary material for: Ecological Patterns of Gut Microbiota During Elexacaftor/Tezacaftor/Ivacaftor Therapy in Cystic Fibrosis
Source: Microorganisms. 2026 Sep 14;14(9):2053. doi: 10.3390/microorganisms14092053 (PMC13608949; doi:10.3390/microorganisms14092053)
Supplement: Supplementary file 1 [file microorganisms-14-02053-s001.zip › microorganisms-4509858-supplementary.pdf]

Supplementary Materials: Tables and Figures

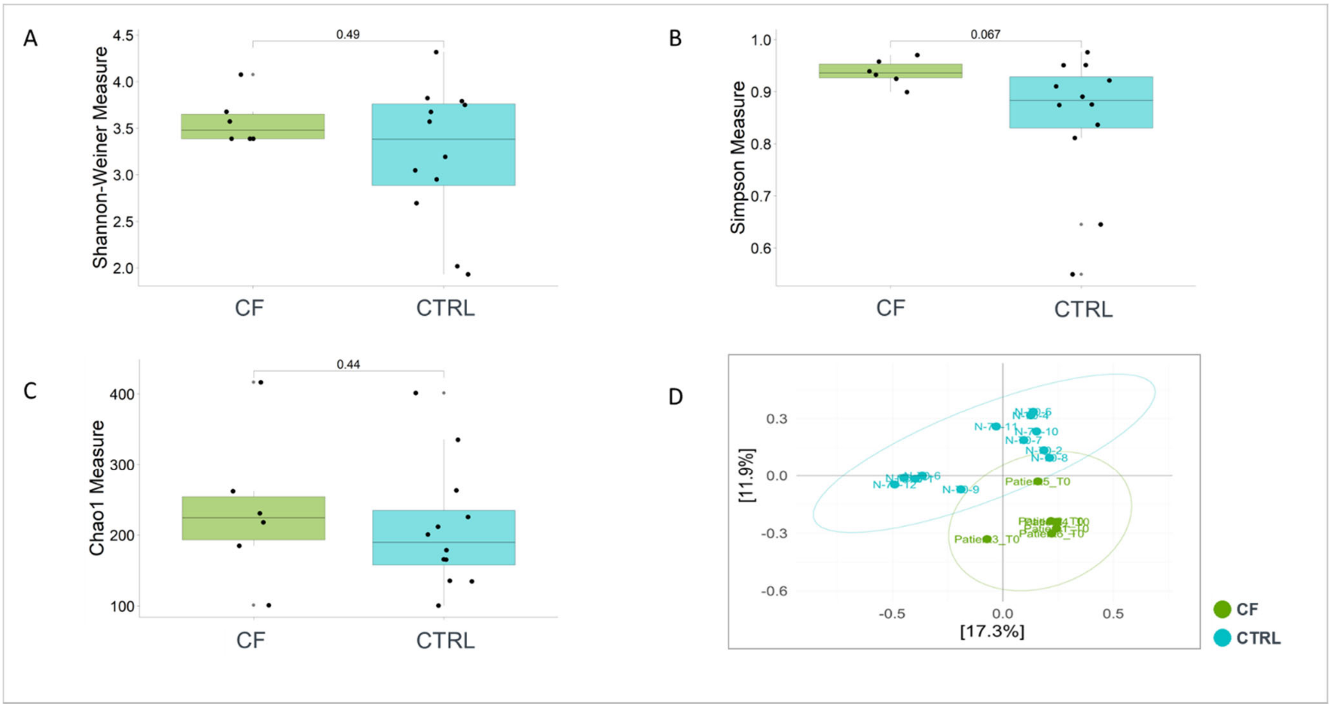

Supplementary Figure S1. Ecological comparison between CF patients at baseline (T0) and the healthy controls (CTRLs). No significant differences in alpha diversity were observed by exploiting the Shannon–Weiner, Simpson, and Chao1 indices **(A-C)**. Similarly, beta diversity, based on Bray–Curtis distance, did not reveal a significant separation between groups in the PCoA plot **(D)** (PERMANOVA: $R^2 = 0.0542$, p-value > 0.05; ANOSIM: R = -0.09, p-value > 0.05).

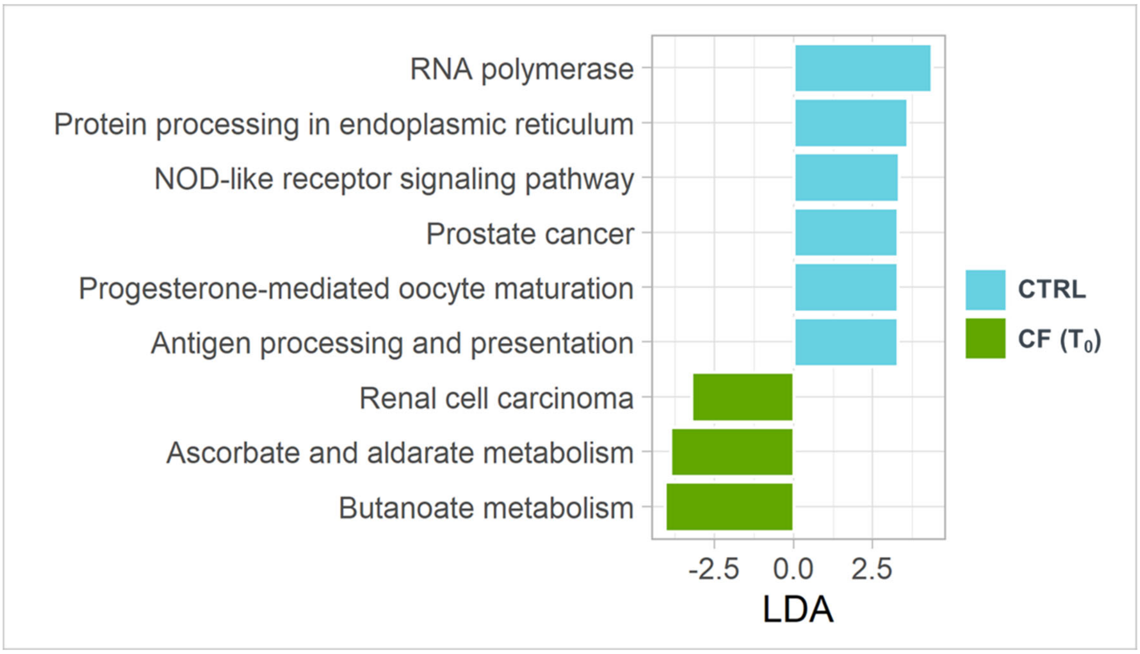

Supplementary Figure S2. **Functional prediction identified metabolic pathways specific to CF patients before treatment (T0), compared with healthy controls (CTRL)**. Functional prediction was performed to identify distinct pathway signatures assigned to pwCF at T0 (green) and CTRL group (blue) using PICRUSt2 and KEGG pathway annotation. Statistics was carried out by applying LEfSe analysis (LDA > 3).

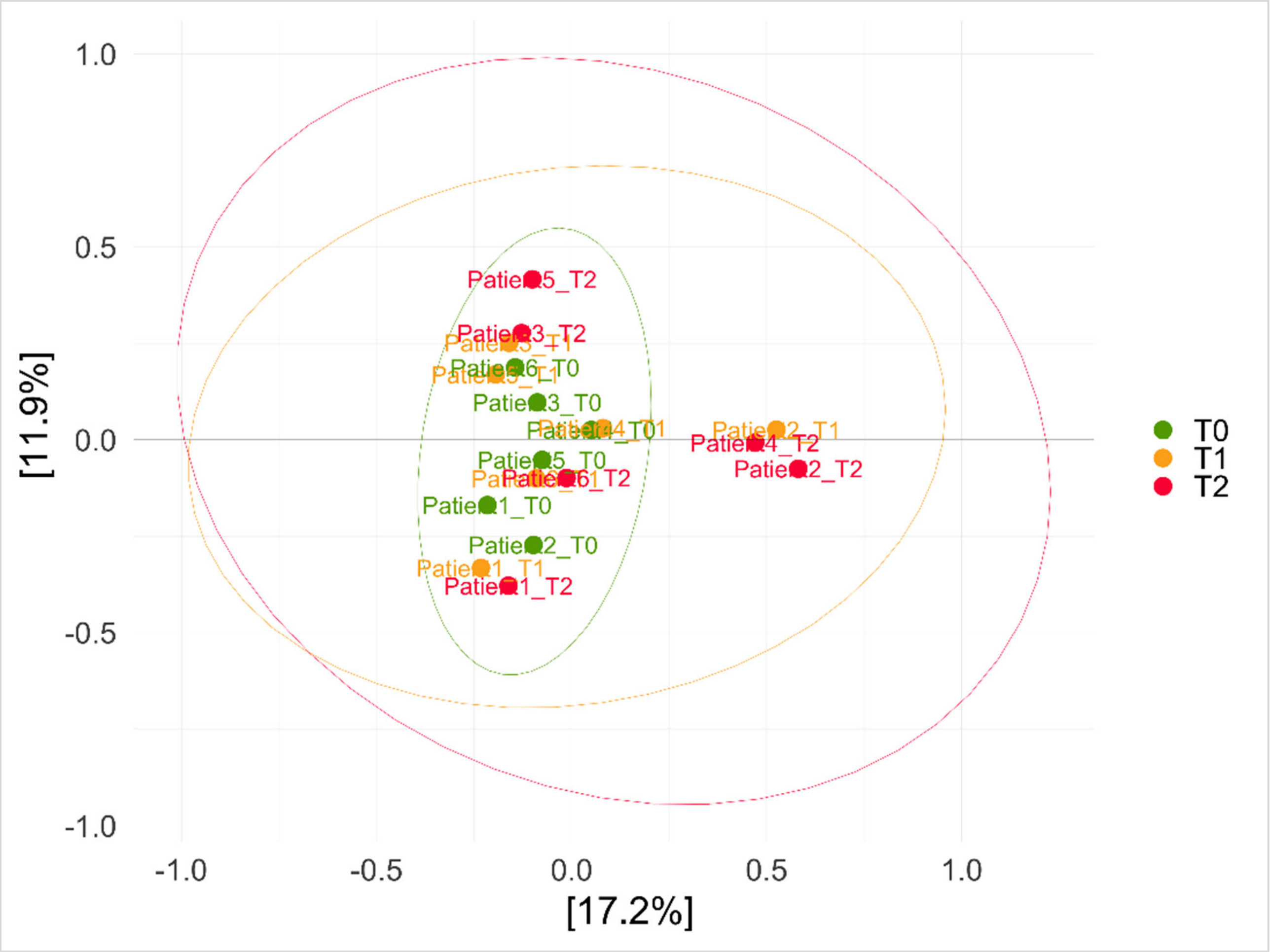

Supplementary Figure S3. Longitudinal comparison of gut microbiota profiles among the T0, T1, and T2 time points in CF patients treated with ETI. Beta diversity was assessed using the Bray–Curtis dissimilarity matrix and visualized by PCoA.

Supplementary Table S1. Values of Friedman and Wilcoxon signed-rank tests performed on GM alpha diversity indices of CF patients at the T0, T1, and T2 time points to assess longitudinal and related pairwise comparisons.

Index	Test	Statistics	*p*-value
Shannon–Weiner	Friedman (T0 vs T1 vs T2)	$\chi^2 = 1.000$	0.607
Shannon–Weiner	Wilcoxon T0 vs T1	W = 10	1
Shannon–Weiner	Wilcoxon T0 vs T2	W = 1	0.0625
Shannon–Weiner	Wilcoxon T1 vs T2	W = 7	0.5625
Chao1	Friedman (T0 vs T1 vs T2)	$\chi^2 = 2.333$	0.311
Chao1	Wilcoxon T0 vs T1	W = 10	1
Chao1	Wilcoxon T0 vs T2	W = 4	0.2188
Chao1	Wilcoxon T1 vs T2	W = 5	0.3125

	T0			T1			T2		
**Index**	**Mean**	**Sd**	**SEM**	**Mean**	**Sd**	**SEM**	**Mean**	**Sd**	**SEM**
Shannon–Weiner	3.58	0.27	0.11	3.19	0.97	0.40	2.89	0.67	0.27
Chao1	235.69	104.34	42.60	235.73	132.76	54.20	162.57	75.43	30.79
